# Preliminary Development and Testing of C595 Radioimmunoconjugates for Targeting MUC1 Cancer Epitopes in Pancreatic Ductal Adenocarcinoma

**DOI:** 10.3390/cells11192983

**Published:** 2022-09-24

**Authors:** Ashleigh Hull, Yanrui Li, Dylan Bartholomeusz, William Hsieh, William Tieu, Tara L. Pukala, Alexander H. Staudacher, Eva Bezak

**Affiliations:** 1 Allied Health and Human Performance Academic Unit, Cancer Research Institute, University of South Australia, Adelaide, SA 5001, Australia; 2 Department of PET, Nuclear Medicine & Bone Densitometry, Royal Adelaide Hospital, SA Medical Imaging, Adelaide, SA 5000, Australia; 3 Adelaide Medical School, The University of Adelaide, Adelaide, SA 5000, Australia; 4 Molecular Imaging and Therapy Research Unit, South Australian Health and Medical Research Institute, Adelaide, SA 5000, Australia; 5 School of Physical Sciences, The University of Adelaide, Adelaide, SA 5000, Australia; 6 Translational Oncology Laboratory, Centre for Cancer Biology, SA Pathology and University of South Australia, Adelaide, SA 5000, Australia

**Keywords:** pancreatic cancer, radioimmunoconjugates, mucin 1, C595

## Abstract

Mucin 1 is a transmembrane glycoprotein which overexpresses cancer-specific epitopes (MUC1-CE) on pancreatic ductal adenocarcinoma (PDAC) cells. As PDAC is a low survival and highly aggressive malignancy, developing radioimmunoconjugates capable of targeting MUC1-CE could lead to improvements in PDAC outcomes. The aim of this study was to develop and perform preliminary testing of diagnostic and therapeutic radioimmunoconjugates for PDAC using an anti-MUC1 antibody, C595. Firstly, p-SCN-Bn-DOTA was conjugated to the C595 antibody to form a DOTA-C595 immunoconjugate. The stability and binding affinity of the DOTA-C595 conjugate was evaluated using mass spectrometry and ELISA. DOTA-C595 was radiolabelled to Copper-64, Lutetium-177, Gallium-68 and Technetium-99m to form novel radioimmunoconjugates. Cell binding assays were performed in PANC-1 (strong MUC1-CE expression) and AsPC-1 (weak MUC1-CE expression) cell lines using ^64^Cu-DOTA-C595 and ^177^Lu-DOTA-C595. An optimal molar ratio of 4:1 DOTA groups per C595 molecule was obtained from the conjugation process. DOTA-C595 labelled to Copper-64, Lutetium-177, and Technetium-99m with high efficiency, although the Gallium-68 labelling was low. ^177^Lu-DOTA-C595 demonstrated high cellular binding to the PANC-1 cell lines which was significantly greater than AsPC-1 binding at concentrations exceeding 100 nM (*p* < 0.05). ^64^Cu-DOTA-C595 showed similar binding to the PANC-1 and AsPC-1 cells with no significant differences observed between cell lines (*p* > 0.05). The high cellular binding of ^177^Lu-DOTA-C595 to MUC1-CE positive cell lines suggests promise as a therapeutic radioimmunoconjugate against PDAC while further work is required to harness the potential of ^64^Cu-DOTA-C595 as a diagnostic radioimmunoconjugate.

## 1. Introduction

Pancreatic ductal adenocarcinoma (PDAC) is the most common form of pancreatic cancer, accounting for up to 90% of pancreatic neoplasms [1,2]. Despite representing the 8th most common cancer diagnosis in the United States of America and Australia, PDAC is the 4th most common cause of cancer-related death and has a 5-year survival rate of 11% [1,3]. The non-specific symptoms, limited early detection methods and late presentation contribute to the poor outcomes [4,5]. Current therapeutic interventions are ineffective against late-stage disease [6,7] and highlight the need for new diagnostic and therapeutic measures capable of targeting advanced PDAC.

Radioimmunoconjugates, formed by binding radionuclides to monoclonal antibodies (mAb), can provide specific targeting of cancer cells to both diagnose and treat diseases [8]. For diagnostic purposes, short-lived radionuclides such as Gallium-68 (^68^Ga), Copper-64 (^64^Cu) and Technetium-99m (^99m^Tc) (Table 1) can deliver non-invasive molecular imaging using positron emission tomography (PET) or single photon emission computed tomography (SPECT). High energy radionuclides with longer half-lives and cytotoxic particle-emissions, including Lutetium-177 (^177^Lu) and Actinium-225 (^225^Ac), can be used for radioimmunotherapy. When diagnostic and therapeutic conjugates target the same cellular receptor or molecular pathway, they can be considered theranostic. In theranostics, the imaging agent identifies if a patient’s disease has sufficient expression of the target receptor to receive the therapeutic agent. This process provides a personalised form of medicine and can inform clinical decisions by predicting the potential therapeutic efficacy and treatment-related toxicities of the radioimmunotherapy based on the individual’s expression profile [9]. The use of theranostics is increasing and has demonstrated value in several cancers, including prostate cancers and neuroendocrine tumours using ^68^Ga/^177^Lu-labelled PSMA and DOTA-TATE, respectively [10,11,12], however success does rely on selection of the target receptor [13].

Mucin 1 (MUC1) is a polymorphic glycoprotein which has a role in cell signalling [14,15]. MUC1 has ubiquitous tissue expression and is physiologically expressed on the apical surface of epithelial tissues, however, it is also aberrantly overexpressed and hypoglycosylated on cancerous cells [16,17,18,19]. This hypoglycosylation exposes the variable number tandem repeat region (VNTR) which holds a number of cancer-specific MUC1 epitopes (MUC1-CE). Several studies have identified these MUC1-CE as having an optimal expression profile for targeted therapy of PDAC [20,21,22,23]. The expression of MUC1-CE has also been correlated with more advanced PDAC, suggesting these epitopes could be used to characterise and treat late-stage disease [24]. Of note is the Arg-Pro-Ala-Pro MUC1-CE which can be targeted by C595, an IgG3 murine mAb [25,26,27]. Over 90% of PDAC overexpress the C595-reactive MUC1-CE yet expression on normal tissues is minimal [20,21,22]. This expression profile is ideal for targeted therapy as it allows for specific targeting of only cancer cells to optimise the therapeutic efficacy and minimise normal-tissue toxicities [28].

Given the optimal expression profile of MUC1-CE, developing complementary radioimmunoconjugates based on the C595 mAb may provide an avenue to detect and treat advanced PDAC. The aim of this study was to develop a novel C595 immunoconjugate and test its radiolabelling capacity with several diagnostic and therapeutic radionuclides to identify prospective radioimmunoconjugates for theranostic applications of PDAC.

## 2. Materials and Methods

### 2.1. Antibody, Chelator, Peptides and Other Reagents

The C595 mAb was purchased from Medical Scitec Australia Pty Ltd. (Sydney, Australia) and prepared by QED Bioscience (San Diego, CA, USA). Rabbit anti-mouse IgG HRP was purchased from Sigma-Aldrich (St. Louis, MO, USA). The bifunctional chelator, p-SCN-Bn-DOTA (S-2-(4-Isothiocyanatobenzyl)-1,4,7,10-tetraazacyclododecane tetraacetic acid, MW: 687.8 g/mol) was purchased from Macrocyclics (Dallas, TX, USA) and MUC1 peptide with the sequence GVTSAPDTRPAPGSTAPPA was purchased from GenScript (Piscataway, NJ, USA). All other reagents were purchased from Sigma-Aldrich (St. Louis, MO, USA) unless otherwise stated. 

### 2.2. Conjugation of p-SCN-Bn-DOTA to C595 mAb

The C595 mAb was buffer exchanged into metal-free phosphate-buffered saline (PBS) using a 50 kDa Amicon^®^ centrifugal filter unit (Merck Milipore, Burlington, MA, USA) to remove sodium azide. To assess the influence of different molar ratios on conjugation, purified C595 (1 mg) was incubated with 20-, 40-, 50- and 100-fold molar excesses of p-SCN-Bn-DOTA in a 0.1 M sodium bicarbonate buffer (pH 8.5) for 2 h at 37 °C in a ThermoMixer^®^ (Eppendorf, Hamburg, Germany) at 700 RPM. The resulting DOTA-C595 immunoconjugate were purified to remove unconjugated DOTA molecules using a 50 kDA Amicon^®^ centrifugal filter unit and buffer transferred into 0.1 M ammonium acetate pH 7.4.

### 2.3. Determination of Number of Chelators per Antibody

The protein concentration of DOTA-C595 conjugate was quantified using Pierce™ BCA Protein Assay Kit (Thermo Fisher Scientific, Waltham, MA, USA) following manufacturer’s instructions. A spectroscopic Cu(II)-Arsenazo(III) assay was performed to quantify the number of p-SCN-Bn-DOTA groups in the conjugate [29]. Briefly, p-SCN-Bn-DOTA stock was diluted to 3 mM in milli-Q water. In triplicate, the 3 mM DOTA stock (10 µL) was serially diluted in 10 µL of milli-Q water in a 96-well plate to produce a standard DOTA curve. Triplicate samples of the purified immunoconjugate (10 µL) were added to separate wells in the well-plate. 1× Cu(II)-Arsenazo(III) solution (190 µL) was added to each well and the well-plate was incubated for 30 min at 37 °C. The absorbance at 630 nm was measured using a FLUOstar Omega (BMG Labtech, Ortenberg, Germany) plate reader. The concentration of p-SCN-Bn-DOTA in the immunoconjugate was calculated using the standard DOTA curve (Supplementary Figure S1). The molar ratio between the DOTA and C595 protein concentrations in the immunoconjugate was then established.

### 2.4. Location of p-SCN-Bn-DOTA Binding on C595 Antibody Chains

The conjugation site of p-SCN-Bn-DOTA to the C595 mAb was determined using mass spectrometry (MS). Samples of DOTA-C595 conjugate and unmodified C595 were buffer exchanged into 0.2 M ammonium acetate using a 50 kDa Amicon^®^ centrifugal filter unit and diluted to 3 mg/mL. Samples were reduced into light and heavy antibody chains through 1:10 addition of 0.1 M of dithiothreitol (DTT) prepared in 0.2 M ammonium acetate. The samples were incubated with DTT at 37 °C for up to 60 min before reading analysis by MS. Protein mass measurements were carried out under denaturing conditions using an Agilent 6230 time-of-flight instrument coupled to an Agilent 1260 Infinity II LC System (Agilent Technologies, Santa Clara, CA, USA). Protein sample (5 µL) was injected and electrosprayed using 50% aqueous acetonitrile/0.01% formic acid at a flow rate of 0.2 mL/min, without chromatographic separation. ESI-MS conditions were: positive-ion mode; capillary voltage, 4000 V; nozzle voltage 2000 V; fragmentor, 200 V; gas 13 L/min; gas temperature, 325 °C; sheath gas 11 L/min; and sheath gas temperature, 350 °C. Spectra were deconvoluted using BioConfirm software (Agilent Technologies, Santa Clara, CA, USA).

### 2.5. Stability of Immunoconjugate Using Mass Spectrometry

To assess DOTA-C595 stability under different storage and physiological conditions, 3 mg/mL samples of the immunoconjugate were stored in conditions consisting of: 0.1 M ammonium acetate pH 7.4 at 4 °C, 0.1 M ammonium acetate pH 7.4 at 37 °C, 0.1 M ammonium acetate pH 6.4 at 37 °C or PBS pH 7.4 at 4 °C. The 4 and 37 °C temperatures were used to represent the typical storage and radiolabelling conditions of the immunoconjugates whilst pH 7.4 and 6.4 were considered to represent the physiological pH and more acidic radiolabelling conditions, respectively. For each storage condition, samples were frozen at baseline (day 0) and every 24 h for 7 days. Additional samples stored in 0.1 M ammonium acetate (pH 7.4) at 4 °C and PBS (pH 7.4) at 4 °C were also assessed at day 30 to assess longer-term stability. On the day of analysis, the samples were defrosted, reduced into heavy and light chains, and prepared for MS using the steps outlined above.

### 2.6. Comparison of Binding Affinity

An ELISA was performed to assess for differences in the binding affinity of unmodified C595 and the DOTA-C595 conjugate to the MUC1-CE. Initially, a 96-well high binding ELISA plate was coated with 20 µg/mL of MUC1 peptide diluted in 0.1 M PBS at pH 7.4 and incubated overnight at 4 °C. The following day, the plate was washed four times using filtered PBS to remove unbound peptide. Non-specific binding was blocked by incubating each well with 150 µL of 3% bovine serum albumin (BSA)/PBS for 1 h at 37 °C. Excess BSA/PBS was removed from the wells following incubation. Serial dilutions (1:2) of 100 µg/mL of DOTA-C595 and unmodified C595 in 2% BSA/PBS were then added to the wells in triplicate samples and incubated for 2 h at 37 °C. Following incubation, the wells were washed with filtered PBS. The wells were then incubated with 100 µL of rabbit anti-mouse IgG HRP diluted in 1% BSA/PBS for 1 h at room temperature. Each well was washed five times using PBS. ABTS substrate was reconstituted by dissolving 1 tablet (10 mg) in 100 mL of 0.05 M phosphate-citrate buffer at pH 5.0 before 100 µL of 30% hydrogen peroxide was added. 100 µL of the ABTS substrate solution was then added to each well and developed for 5 min at room temperature. Absorbance at 405 nm was measured at 30 min with background readings subtracted. Dissociation constants (KD) values were determined using GraphPad Prism (v. 8, GraphPad Software Inc., San Diego, CA, USA).

### 2.7. Radiolabelling of DOTA-C595

Fresh DOTA-C595 was radiolabelled with ^99m^Tc, ^177^Lu, ^68^Ga and ^64^Cu. Briefly for ^99m^Tc labelling, 0.1 mg of stannous chloride (30 µL of 3.33 mg/mL) was added to a vial containing 500 µL of 10% acetic acid. 100 µg of DOTA-C595 was added to the vial followed by 200 MBq of freshly eluted ^99m^Tc. Sodium bicarbonate (2 mL) was also added to achieve a pH of 7. The reaction vial was incubated at room temperature for 2 h. ^177^Lu-DOTA-C595 was produced by adding 40 MBq of ^177^LuCl_3_ and 500 µg of DOTA-C595 to 100 µL of 0.5 M ammonium acetate pH 6. The reaction was incubated at 37 °C for 1 h using a ThermoMixer^®^ set to 500 RPM. To label with ^68^Ga, 100 µg of DOTA-C595 was added to a vial containing 0.2 mL of 1 M sodium acetate trihydrate pH 4, followed by the addition of 100 MBq of freshly eluted ^68^Ga in 1 mL of 0.1 M hydrochloric acid. The reaction was incubated at 37 °C for 30 min then purified using 10 kDa Amicon^®^ centrifugal filter unit. Labelling with ^64^Cu involved incubating 20 MBq of ^64^Cu with 240 µg of DOTA-C595 in a reaction vial containing 100 µL of 0.1 M ammonium acetate, pH 6. The reaction vial was incubated at 37 °C for 30 min on a ThermoMixer^®^ set to shake at 600 RPM.

### 2.8. Determination of Radiolabelling Yield and Purity

Instant-thin layer chromatography (ITLC) was used to monitor the reactions and determine radiolabelling yield. For each radioimmunoconjugate, a 3 µL sample of the reaction solution was aliquoted onto a silica-gel ITLC (ITLC-SG) strip. The ^99m^Tc and ^177^Lu strips were developed in acetone and 25 mM DTPA pH 6, respectively. Both ^68^Ga and ^64^Cu strips were developed in 0.1 M citric acid pH 5. The ITLC strip was scanned using a Scan-Ram radio TLC scanner (LabLogic Systems Ltd., Tampa, FL, USA).

Radiochemical purity and protein quantification of the ^177^Lu and ^64^Cu radioimmunoconjugates was evaluated using Size Exclusion Chromatography-High Performance Liquid Chromatography (SEC-HPLC). Analysis was performed on a Shimadzu LC-20 series with SPD-20A detector, using Agilent AdvanceBio SEC 300 (2.7 µm, 300 × 7.4 mm) column with PBS buffer (pH 6.8) at a flowrate of 1 mL/min. 30 µL of sample were injected. Protein quantification was determined by area under the curve of major peak (5–7 min) and was based on standard curve of known mAb concentration (4.8 mg/mL) at 220 nm and 280 nm.

### 2.9. Cell Cultures

The human pancreatic cancer cell lines PANC-1 and AsPC-1 were purchased from American Type Culture Collection (Manassas, VA, USA). PANC-1 cells were cultured using Dulbecco’s Modified Eagle Medium (DMEM) supplemented with 10% foetal bovine serum (FBS) and 1% penicillin/streptomycin (P/S). AsPC-1 cells were cultured using Roswell Park Memorial Institute (RPMI) 1640 medium supplemented with 10% FBS and 1% P/S. All cell lines were grown in T75 flasks and incubated at 37 °C with 5% carbon dioxide in atmospheric oxygen. Cells were used within three months of resuscitation and regularly monitored for mycoplasma contamination using MycoAlert detection kit (Lonza Group Ltd., Basel, Switzerland). Unless otherwise stated, all cell culture reagents were purchased from Sigma-Aldrich Pty Ltd. (Castle Hill, Australia).

For experiments, cells were grown to confluence then washed twice with PBS and detached from the flask by TrypLE™ Select Enzyme (1X) (Thermo Fisher Scientific Australia Pty Ltd., Scoresby, Australia). Detached cells were centrifuged at 300× *g* for 5 min to separate the cell pellet and supernatant to prepare cells for seeding.

### 2.10. Cell Binding Assays

To evaluate the binding capacity of the radioimmunoconjugates to MUC1-CE, separate cell binding assays were performed using ^177^Lu-DOTA-C595 and ^64^Cu-DOTA-C595. Our previous work has identified that 93.1% of PANC-1 cells express MUC1-CE compared to only 11.5% of AsPC-1 cells (Supplementary Figure S2) [22]. Due to the variable MUC1-CE expression, these cell lines were used to evaluate for binding differences relative to MUC1-CE expression. Briefly, PANC-1 and AsPC-1 cells (10^5^ cells suspended in 250 µL of media) were seeded into 24-well plates and incubated for 48 h at 37 °C. Varying concentrations of radioimmunoconjugate (0–500 nM) were added to the cells in triplicate and incubated for 1 h at 37 °C. Following incubation, excess media and unbound radioimmunoconjugate were removed from the wells. Cells were washed twice using PBS then detached using TrypLE™ Select Enzyme (1X). The cell lysates were collected and the counts per minute (CPM) of each cell sample was measured using a Hidex Automatic Gamma Counter (Hidex, Turku, Finland). Outliers were removed and background-corrected total cell binding in CPM was plotted against radioimmunoconjugate concentration. The graph was fitted with non-linear least squares regression. Nonspecific binding was considered as proportional to the radioimmunoconjugate concentration.

A separate blocking experiment was also performed using ^64^Cu-DOTA-C595. In this experiment, MUC1-CE were blocked by incubating cells with unlabelled C595 for 1 h at 4 °C before proceeding with the cell binding experiment as detailed above.

### 2.11. Influence of Storage on Radiolabelling and Cell Binding

To determine if DOTA-C595 remained stable after long-term storage, the radiolabelling and cell binding experiments were repeated using ^177^Lu and ^64^Cu. For the repeated ^177^Lu experiments, the conjugate was stored in a 0.1 M ammonium acetate buffer pH 7 at 4 °C for 120 days before experiments were repeated. The storage time was extended to 180 days for the repeated ^64^Cu experiments. The delayed radiolabelling and cell binding assays were compared to the original ^177^Lu and ^64^Cu results achieved when using fresh DOTA-C595.

### 2.12. Statistical Analysis

All statistical analyses were performed using GraphPad Prism (v. 8, GraphPad Software Inc., San Diego, CA, USA). Unless otherwise stated, all experiments were performed in triplicate with data presented as mean ± standard deviation. *p*-values for cell binding data were calculated by performing multiple paired *t*-tests. Significance was deemed when *p* < 0.05.

### 2.13. Facilities

All non-radioactive experimental procedures were conducted at the Cancer Research Institute, University of South Australia. Mass spectrometry was performed at the School of Physical Sciences, The University of Adelaide. Radiolabelling and quality control tests were performed in licensed radiation laboratories at the Department of PET, Nuclear Medicine and Bone Densitometry, Royal Adelaide Hospital and the Molecular Imaging and Therapy Research Unit (MITRU), South Australian Health and Medical Research Institute (SAHMRI). Cell binding assays using the radioimmunoconjugates were also performed at MITRU, SAHMRI.

## 3. Results

### 3.1. Conjugation of p-SCN-Bn-DOTA to C595

The average number of chelators attached per antibody increased with the molar excess of p-SCN-Bn-DOTA added (Table 2). An average chelator-to-antibody ratio of 4:1 was selected as an appropriate starting point for radiolabelling for two reasons. Firstly, chelator-to-antibody ratios exceeding 5 have previously been shown to alter the biochemical properties of the conjugate and decrease immunoreactivity of the antibody [29]. Secondly, low chelator-to-antibody ratios can limit the specific activities which can be radiolabelled [30]. As such, an average chelator-to-antibody ratio of 4:1 was considered an optimal middle point for radiolabelling at appropriate specific activity for cell-based studies without disrupting the immunoreactivity of the antibody. Therefore, for all remaining experiments, the DOTA-C595 conjugate was produced using a 40-fold molar excess of p-SCN-Bn-DOTA.

**Figure 1:**
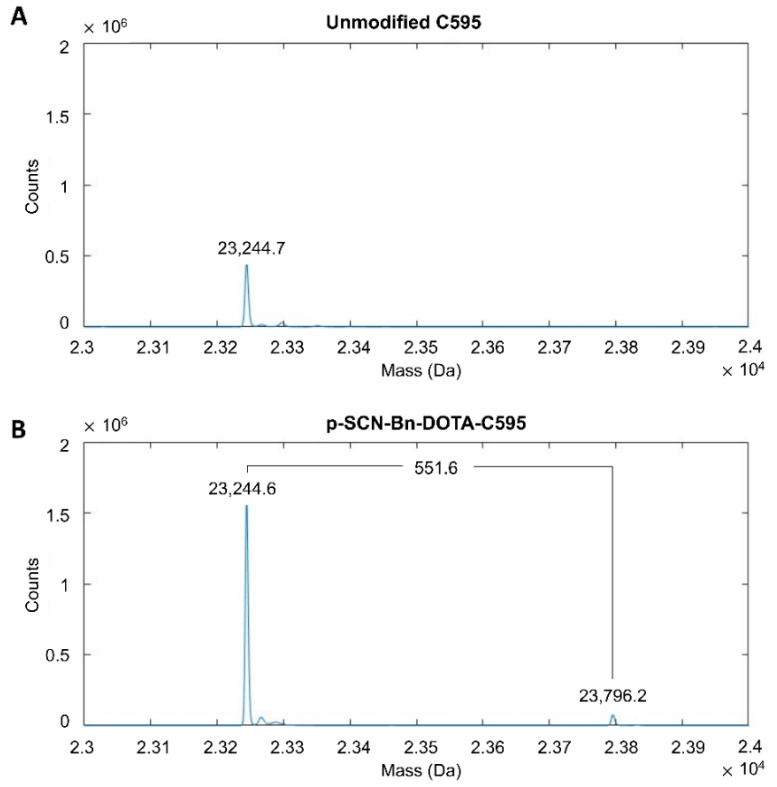
Mass spectra of the light chains of (**A**) unmodified C595 and (**B**) p-SCN-Bn-DOTA-C595 conjugate.

**Figure 2:**
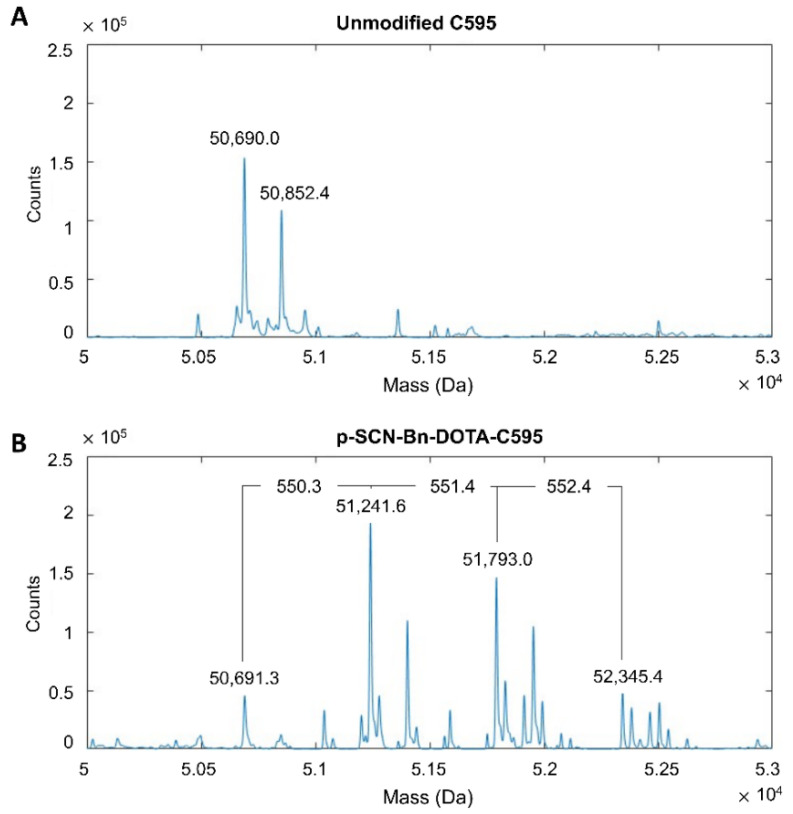
Mass spectra of the heavy chains of (**A**), unmodified C595 and (**B**), p-SCN-Bn-DOTA-C595 conjugate.

The presence of p-SCN-Bn-DOTA binding to the C595 antibody chains was indicated by a mass shift of approximately 551 Da measured by MS. When reduced into light and heavy chains, unmodified C595 demonstrated primary mass peaks of 23,244.7 Da and 50,690.0 Da, respectively (Figure 1A and Figure 2A). An additional mass peak was noted on the light chain of the DOTA-C595 conjugate at 23,796.2 Da, representing a 551.6 Da mass shift equal to the binding of a single DOTA molecule (Figure 1B). Three additional mass peaks were identified on the heavy chain of the DOTA-C595 conjugate at 51,241.6 Da (mass shift of 550.3 Da), 51,793.0 Da (mass shift of 551.4 Da) and 52,345.4 Da (mass shift of 552.4 Da) (Figure 2B). These findings indicate preferential binding of p-SCN-Bn-DOTA to the heavy C595 chain and confirmed a maximum 4:1 molar ratio of DOTA to C595 molecules. Secondary peaks were also identified on the heavy chains and likely indicate variable glycosylation of the C595 mAb.

### 3.2. Stability of DOTA-C595 Immunoconjugate

**Figure 3:**
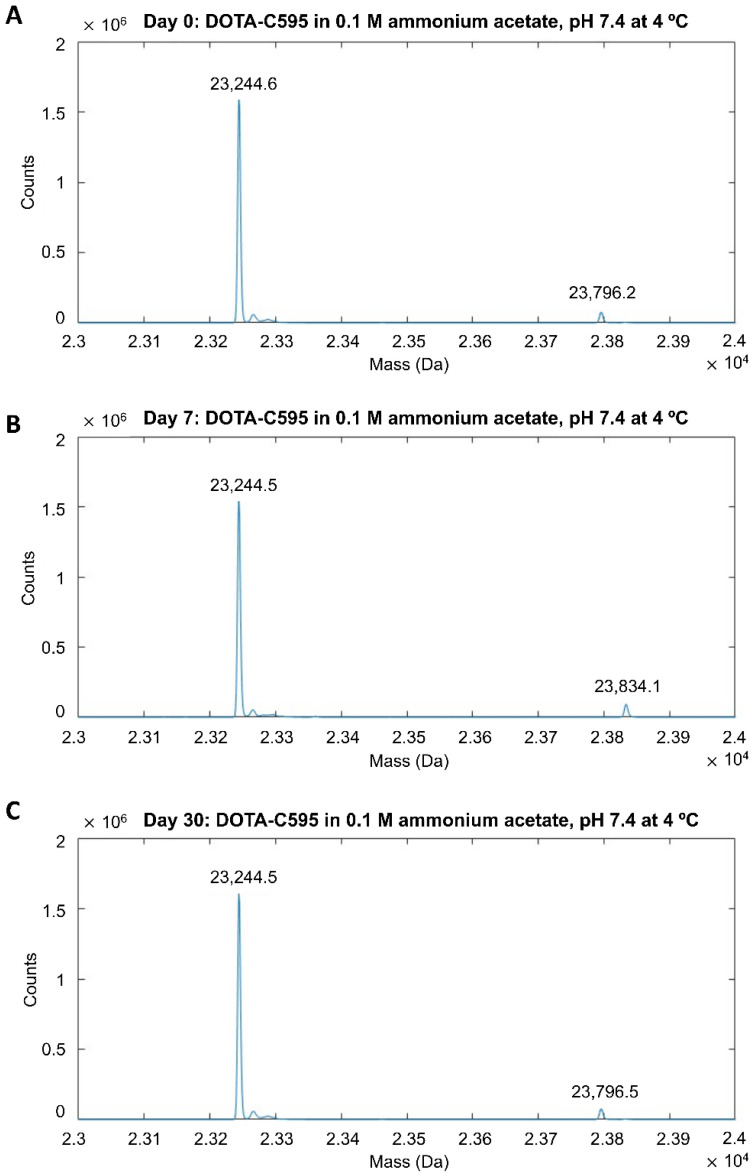
Mass spectra of light chain p-SCN-Bn-DOTA-C595 conjugate stored in 0.1 M ammonium acetate (pH 7.4) at 4 °C at (**A**) day 0, (**B**) day 7 and (**C**) day 30.

**Figure 4:**
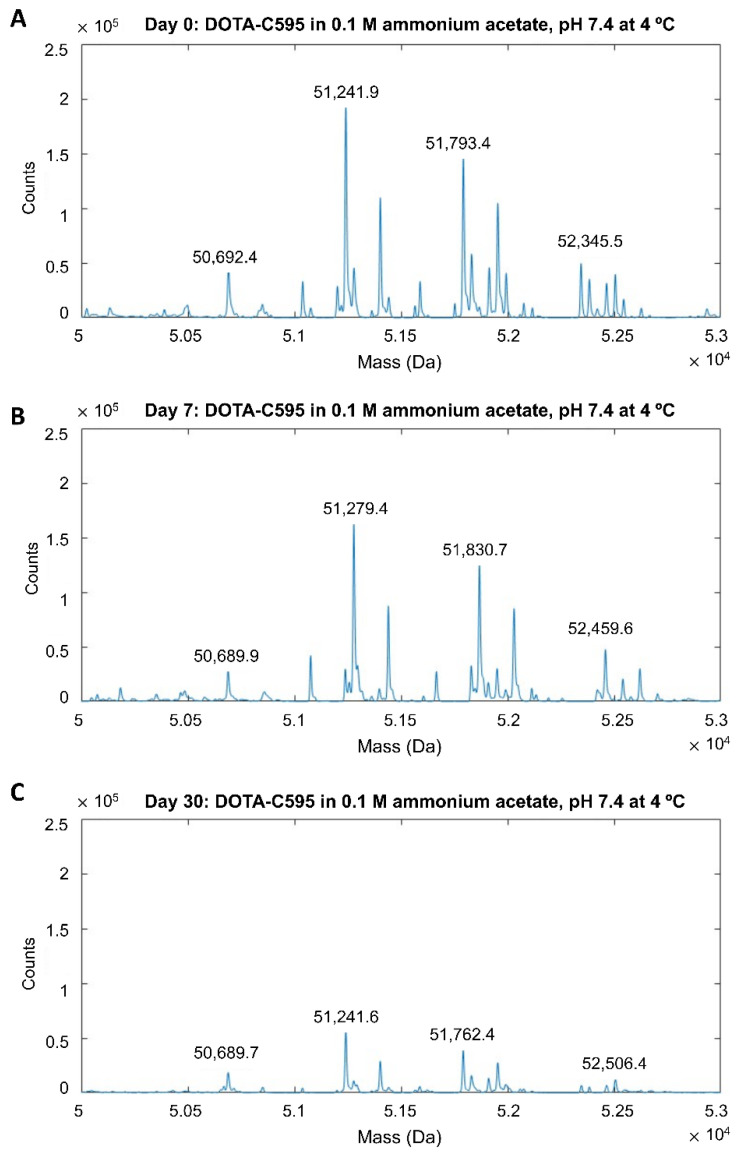
Mass spectra of heavy chain p-SCN-Bn-DOTA-C595 conjugate stored in 0.1 M ammonium acetate (pH 7.4) at 4 °C at (**A**) day 0, (**B**) day 7 and (**C**) day 30.

Mass spectra demonstrated the DOTA-C595 conjugation was stable. The primary storage condition of 0.1 M ammonium acetate pH 7.4 at 4 °C demonstrated similar mass spectra for samples analysed at day 0, 7 and 30 (Figure 3 and Figure 4). On the heavy chain, a reduction in ions counts was observed at day 30 which may suggest possible sample precipitation; however, no count reductions were noted on the light chain. The 4:1 molar ratio also remained stable when the immunoconjugate was stored in 0.1 M ammonium acetate at pH 6.4 or pH 7.4 at 37 °C for 7 days, with minimal differences observed in mass peaks and sample counts, except for low sample counts on the day 7 heavy chain sample stored at pH 7.4 (Supplementary Figures S3–S6). Both the heavy and light chain samples stored in PBS (pH 7.4) at 4 °C demonstrated low ion counts at Day 30 compared to Day 0, however the location of the mass peaks remained stable (Supplementary Figures S7 and S8).

### 3.3. Effect of p-SCN-Bn-DOTA Conjugation to C595 Binding Affinity

**Figure 5:**
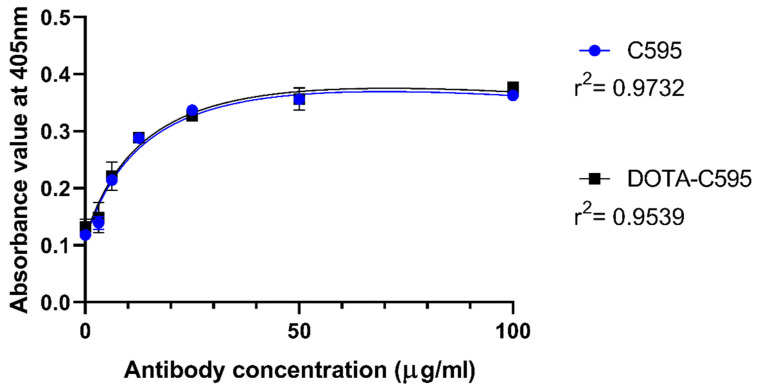
ELISA assay comparing the binding affinity of p-SCN-Bn-DOTA-C595 and unmodified C595 to MUC1 peptide at 405 nm absorbance.

Compared to unmodified C595, DOTA-C595 demonstrated similar binding to the MUC1 peptide at all analysed C595 concentrations (Figure 5). KD values did not significantly differ between unmodified C595 and DOTA-C595 (113 nM vs. 108 nM, *p* = 0.515), confirming the p-SCN-Bn-DOTA conjugation process did not affect C595 binding affinity to MUC1-CE.

### 3.4. Radioimmunoconjugate Production

**Figure 6:**
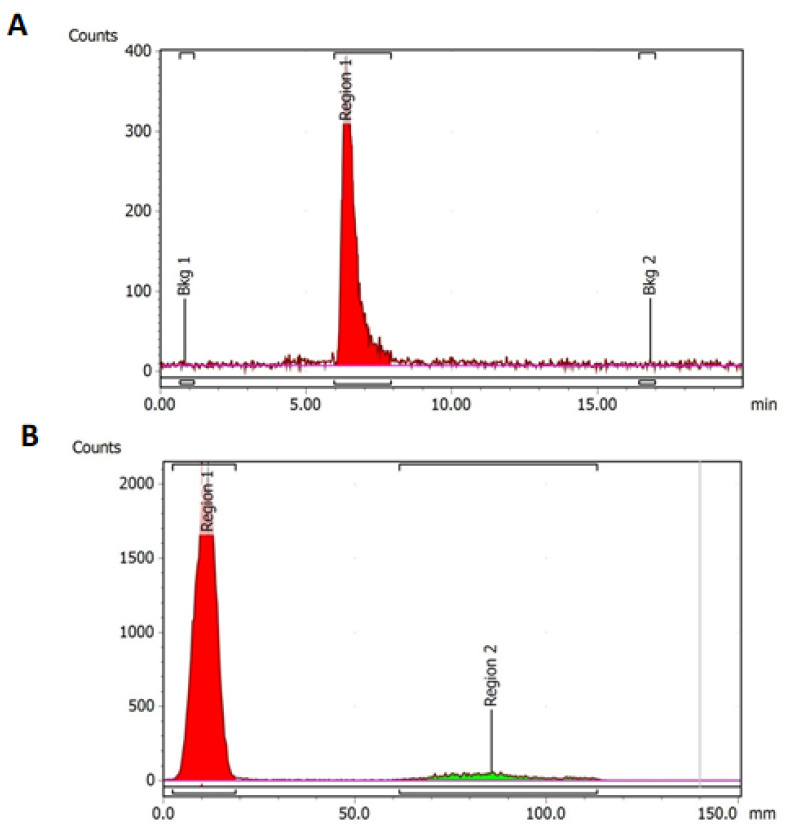
(**A**) HPLC radio-chromatogram of ^64^Cu-DOTA-C595. Region 1 (red) represents ^64^Cu-DOTA-C595 and (**B**) ITLC radio-chromatogram of ^177^Lu-DOTA-C595. Region 1 (red) represents ^177^Lu-DOTA-C595. Region 2 (green) represents free ^177^Lu.

Radiolabelling with ^64^Cu demonstrated the highest labelling yield and radiochemical purity of the labelling reactions in this study (Table 3, Figure 6A). Labelling with ^177^Lu (Figure 6B) and ^99m^Tc also produced high yields exceeding 90% without the need for additional purification, but radiochemical purity was lower compared to ^64^Cu. There was a low radiolabelling yield of ^68^Ga-DOTA-C595 of approximately 30% following the initial 30 min incubation period. Extending the ^68^Ga reaction time to 2 h and purifying the resulting radioimmunoconjugate improved radiolabelling yield, however the yield remained low at only 50% radiolabelling efficiency.

Despite high radiolabelling yield, ^99m^Tc-DOTA-C595 was unable to be analysed using SEC-HPLC. A similar issue was also identified using purified ^68^Ga-DOTA-C595. While these issues may be due to SEC-HPLC column, it may also demonstrate weak bonds between ^99m^Tc, ^68^Ga and DOTA-C595 which were unable to withstand SEC-HPLC pressures.

### 3.5. Cell Binding Assays

Due to the high radiolabelling efficiency, purity and simple preparation methods, cell binding assays were performed using ^64^Cu-DOTA-C595 and ^177^Lu-DOTA-C595.

**Figure 7:**
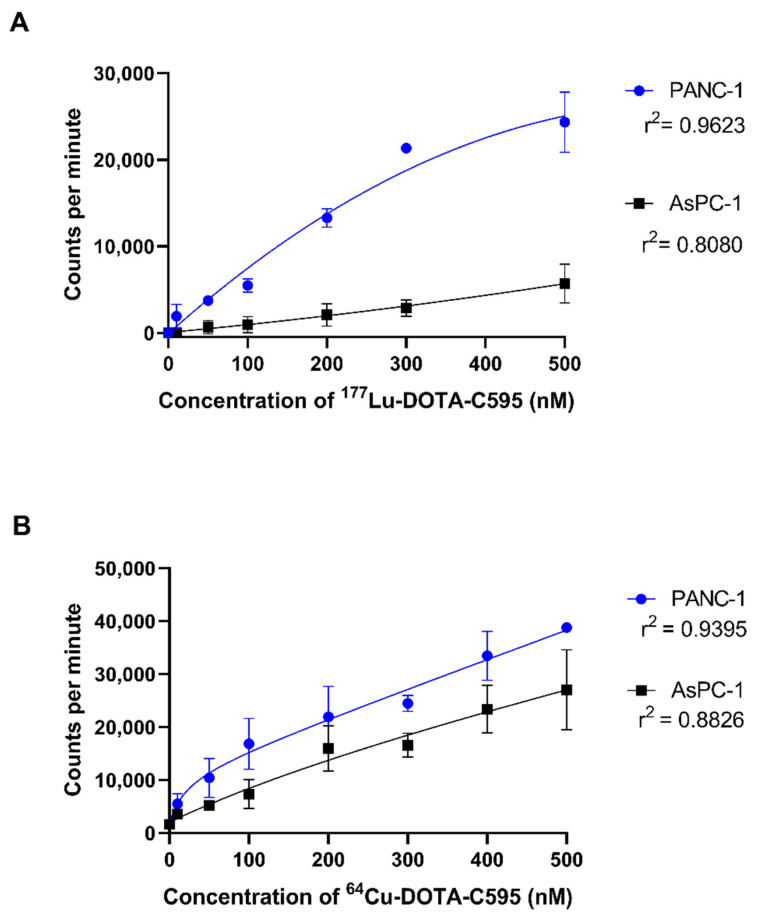
Cell binding (1 × 10^5^ cells) of (**A**) ^177^Lu-DOTA-C595 and (**B**) ^64^Cu-DOTA-C595 to PANC-1 and AsPC-1 cell lines. Data presented as mean ± standard deviation.

In the ^177^Lu-DOTA-C595 experiment, radioimmunoconjugate binding steadily increased in PANC-1 cells with concentration (Figure 7A). Binding to PANC-1 cells saturated at approximately 400 nM of ^177^Lu-DOTA-C595. Only small increases in cell binding were observed in AsPC-1 cells as ^177^Lu-DOTA-C595 concentration increased, with binding also saturating around 400 nM. At concentrations of 100 nM and greater, ^177^Lu-DOTA-C595 binding was significantly increased on PANC-1 cells compared to AsPC-1 cells (*p* < 0.005) (Table 4). This indicates preferential binding of ^177^Lu-DOTA-C595 to the strongly MUC1-CE expressing PANC-1 cell line.

No significant differences were identified between the cellular binding of ^64^Cu-DOTA-C595 to the PANC-1 and AsPC-1 cell lines (Table 4). For both cell lines, ^64^Cu-DOTA-C595 binding followed a similar upward trend as concentration increased. Saturation of ^64^Cu-DOTA-C595 binding was not achieved for PANC-1 or AsPC-1 (Figure 7B).

**Figure 8:**
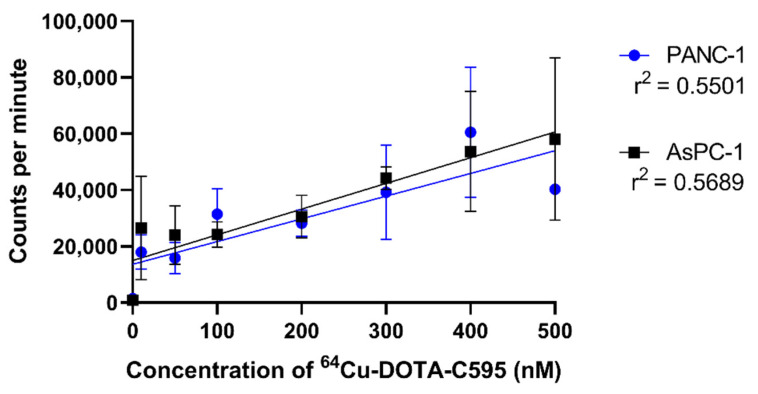
Blocking experiment demonstrating the binding of ^64^Cu-DOTA-C595 to PANC-1 and AsPC-1 cells following C595-blocking. Data presented as mean ± standard deviation.

When PANC-1 and AsPC-1 cells were blocked using C595, the binding of ^64^Cu-DOTA-C595 was linear with concentration. There was minor variation in the binding to both PANC-1 and AsPC-1 cells following the C595 blocking (Figure 8).

### 3.6. Influence of Storage on Radiolabelling and Cell Binding

**Figure 9:**
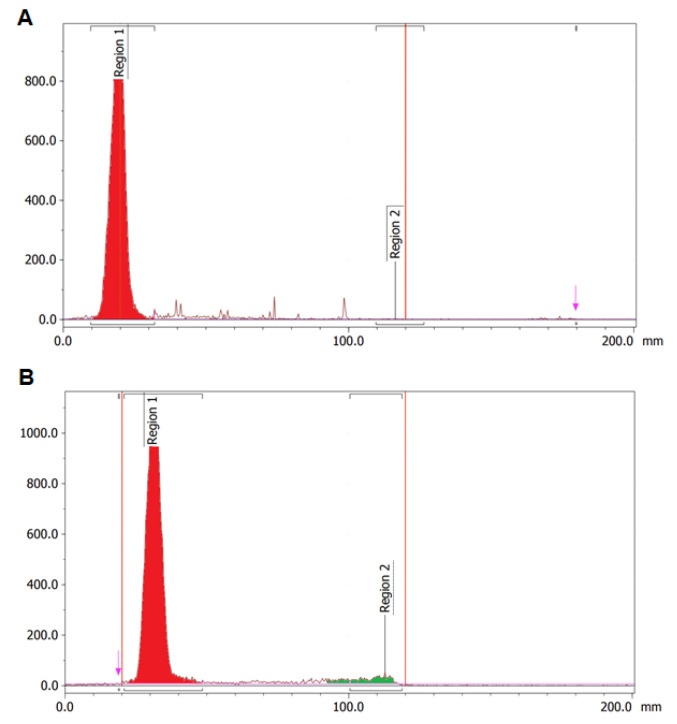
Radiolabelling efficiency using radio-ITLC for radiolabelling of (**A**) ^177^Lu and (**B**) ^64^Cu to 120-day old DOTA-C595.

**Figure 10:**
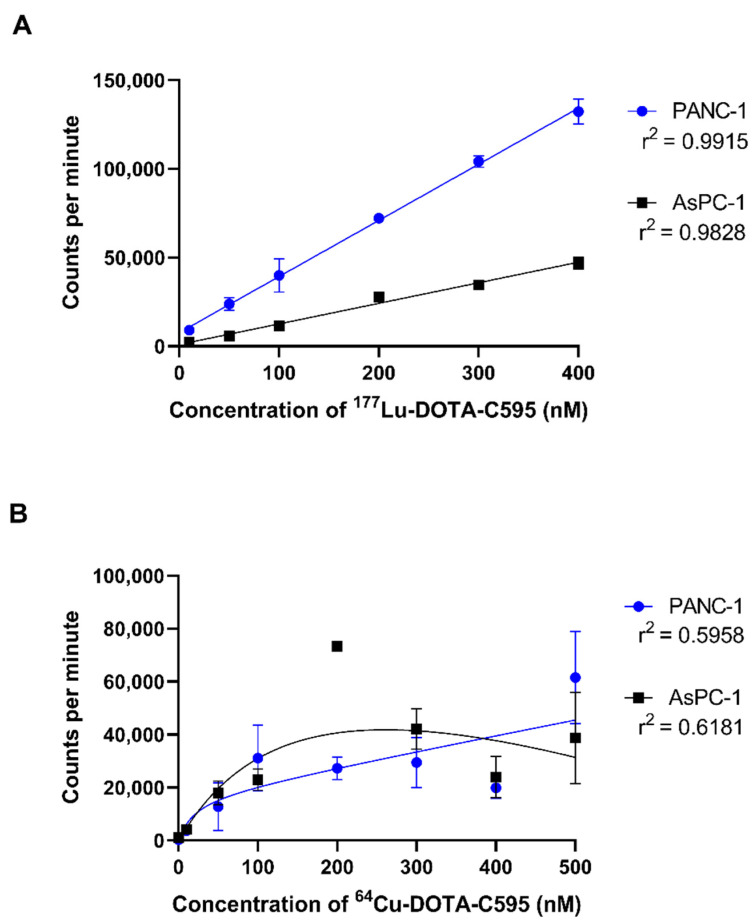
Repeated cell binding assays performed using (**A**) stored ^177^Lu-DOTA-C595 and (**B**) stored ^64^Cu-DOTA-C595. Data presented as mean ± standard deviation.

Both ^177^Lu and ^64^Cu labelled to 120- and 180-day old DOTA-C595, respectively, with high efficiency. There were no significant changes in the radio-ITLC when the radionuclides were labelled to fresh DOTA-C595 compared to the stored DOTA-C595 (Figure 9). The stored ^177^Lu-DOTA-C595 demonstrated a similar binding trend as the fresh ^177^Lu-DOTA-C595 (Figure 10), confirming stability of DOTA-C595 for up to 120 days. Stored ^64^Cu-DOTA-C595 had inconsistent binding to both cell lines which may suggest degradation of the conjugate by 180 days.

## 4. Discussion

Despite minor improvements in survival rates over recent years, PDAC continues to have a poor prognosis and requires new targeted therapies that are effective against the metastatic disease often observed at the time of presentation. Radioimmunotherapy is one method which could provide a highly targeted PDAC treatment. The utility of radioimmunotherapy can be improved by developing complementary imaging radioimmunoconjugates which allow for the informed selection of patients who have optimal expression of the target receptor for radioimmunotherapy [9,12]. We have previously identified MUC1-CE as an ideal target receptor given its expression is correlated with advanced PDAC and it is overexpressed on up to 90% of PDAC cells yet minimally expressed on normal cells [20,21,22]. Effective targeting of MUC1-CE is feasible using the C595 antibody and provides an avenue for radioimmunoconjugate development. In this study, we developed the DOTA-C595 immunoconjugate using the p-SCN-Bn-DOTA chelator and produced four C595-based diagnostic and therapeutic radioimmunoconjugates for potential preclinical and clinical testing in PDAC.

The conjugation of p-SCN-Bn-DOTA to the C595 antibody was performed to produce a universal immunoconjugate for labelling with different radionuclides. P-SCN-Bn-DOTA was the chelator of choice given its aromatic isothiocyanate group which is known to react and remain kinetically inert with most radionuclides [31]. Typically, p-SCN-Bn-DOTA forms covalent bonds with antibodies via the lysine side chains and thiourea coupling [31,32]. There are around 80 lysine residues found on antibodies and due to their polarity and hydrophilicity, many of these are located on the protein surface, making them an easily accessible binding site [33,34]. Our study determined a 3:1 ratio of p-SCN-Bn-DOTA molecules functionalising on the C595 heavy chain compared to light chain. The conjugation process also achieved a maximum molar ratio of 4:1 when a 40-fold molar excess of p-SCN-Bn-DOTA was added. The molar ratio is optimal to ensure sufficient DOTA sites for radiolabelling without largely affecting the C595 binding kinetics of the radioimmunoconjugate and allows estimation of the overall payload achievable by the radioimmunoconjugate [35].

The binding affinity of C595 to MUC1-CE remained unchanged when DOTA-conjugated as demonstrated by the KD values. In this study, the ELISA was performed using a MUC1 peptide with sequence of GVTSAPDTRPAPGSTAPPA which positioned the C595-reactive RPAP section within the middle of the MUC1-CE protein sequence [25,26,27]. The ability of the DOTA-C595 conjugate to bind in the presence of neighbouring amino acids was assessed by centring the target epitope within the sequence, providing more physiological experimental conditions. 

Mass spectra demonstrated the DOTA-C595 immunoconjugate was stable and retained an average 4:1 molar ratio in all storage conditions assessed in this study. Of particular interest was the comparison of long-term storage in either 0.1 M ammonium acetate, pH 7.4 at 4 °C or PBS pH 7.4 at 4 °C. Whilst both long-term storage conditions showed a stable conjugate, low ion counts were noted on the heavy chain samples at day 30. The authors speculate the low counts may be due to antibody degradation from repeated freeze–thaw cycles needed to ensure a single MS session, or the unstable buffering capacity of ammonium acetate at pH 7.4 [36,37]. Whilst the cause of the low ion counts cannot be determined in this study, the authors conclude 0.1 M ammonium acetate, pH 7.4 at 4 °C is the preferred storage condition for the DOTA-C595 immunoconjugate as unlike PBS, ammonium acetate is a commonly used radiolabelling buffer for several radionuclides. Storage of DOTA-C595 in ammonium acetate can then reduce the need for buffer transferring, which will better maintain the integrity of the conjugate. Furthermore, storage of DOTA-C595 in 0.1 M ammonium acetate, pH 7.4 at 4 °C for 120 days was found to have no effect on radiolabelling and binding affinity, as determined by cell binding assays. Extending the storage time to 180 days was shown to impair binding affinity through the likely degradation of the conjugate. Based on these results, it is the recommended to use stored DOTA-C595 within a 120-day window.

In this study, we produced four radioimmunoconjugates—^64^Cu-DOTA-C595, ^177^Lu-DOTA-C595, ^99m^Tc-DOTA-C595 and ^68^Ga-DOTA-C595. All radioimmunoconjugates except for ^68^Ga-DOTA-C595 demonstrated fast and efficient radiolabelling processes and were performed using relatively mild and simple conditions. However, confirmation of radiochemical purity via SEC-HPLC was only possible using ^64^Cu-DOTA-C595 and ^177^Lu-DOTA-C595. The inability to evaluate the radiochemical purity of ^99m^Tc-DOTA-C595 and ^68^Ga-DOTA-C595 may suggest weak bonds between ^99m^Tc/^68^Ga and DOTA-C595 which are unable to withstand SEC-HPLC pressures. Slow reaction kinetics, low thermodynamic stability and the potential for gallium ion hydrolysis likely contributed to the low ^68^Ga radiolabelling yield and purity [38,39]. The former two factors are likely due to the small ionic radii of Ga(III) which is incompatible with the large DOTA coordination cavity (Table 5) [32,40]. Whilst the ionic radius of Tc(IV) is similar to Ga(III), the 6 h half life of ^99m^Tc allows for reactions to be extended to improve radiolabelling yield. Extending ^68^Ga labelling beyond 2 h is not feasible due to the short 68 min half-life. Instead, ^68^Ga to DOTA reactions are typically heated to high temperatures of 90 °C to improve radiolabelling [41,42]. Such temperatures were unable to be used in this study due to the potential for C595 antibody degradation and subsequent impact on the antibodies binding potential [43]. All radiolabelling protocols were thus limited to 37 °C to prevent C595 aggregation.

In terms of cellular binding, ^177^Lu-DOTA-C595 showed significantly greater binding to the PANC-1 cells, which strongly express MUC1-CE, compared to the weakly expressing cell line, AsPC-1. The comparative binding of ^177^Lu-DOTA-C595 between the cell lines suggests the C595 binding kinetics remain intact following ^177^Lu radiolabelling and warrant further investigations into ^177^Lu-DOTA-C595 as a therapeutic radioimmunoconjugate for PDAC. Interestingly, there were no significant differences identified in the binding of ^64^Cu-DOTA-C595 between the two cell lines. Altering the specific activity may allow for this difference to be better identified. It is also speculated that this lack of difference may be due to the antibody being obstructed from accessing the target epitope due to the location of the chelation site and binding geometry of ^64^Cu to the p-SCN-Bn-DOTA molecule; however structural analysis of ^64^Cu-DOTA-C595 is needed to confirm this. Regardless, the binding of ^64^Cu-DOTA-C595 demonstrated an upward trend for both cell lines, suggesting total cell binding increases with concentration.

The difference in cellular binding between ^177^Lu-DOTA-C595 and ^64^Cu-DOTA-C595, as observed in this study, may impair the utility of using these radioimmunoconjugates for theranostic applications. Future work should consider more detailed in vivo investigations to compare the biodistribution of the ^177^Lu-DOTA-C595 and ^64^Cu-DOTA-C595 to further elucidate the theranostic potential against PDAC. 

## 5. Conclusions

We have developed a C595 immunoconjugate for targeting MUC1-CE using the p-SCN-Bn-DOTA chelator. The DOTA-C595 immunoconjugate was optimised in terms of molar ratio, stability and binding affinity. Radiolabelling the DOTA-C595 conjugate with ^64^Cu and ^177^Lu was efficient and highlighted the exceptional radiolabelling capacity of the DOTA-C595 immunoconjugate to different radionuclides. However, radiolabelling to ^99m^Tc and ^68^Ga demonstrated weak radiolabelling capacity which requires further optimisation. Both ^64^Cu-DOTA-C595 and ^177^Lu-DOTA-C595 showed effective cell binding, although only ^177^Lu-DOTA-C595 demonstrated preferential cell binding to MUC1-CE positive cells (*p* < 0.05 at concentrations of 100 nM and greater). These preliminary findings highlight the potential promise of C595-based radioimmunoconjugates for the diagnosis and treatment of PDAC.

## Figures and Tables

**Table 1:** Chemical and physical properties of radionuclides commonly used as radioimmunoconjugates.

Radionuclide	Half-Life (T_1/2_)	Primary Decay Mode and Maximum Energy (MeV)	Clinical Application
^99m^Tc	6 h	*γ* = 0.140	Diagnostic SPECT
^68^Ga	68 min	β+ = 1.89	Diagnostic PET
^177^Lu	6.7 d	*γ* = 0.208; β− = 0.497	Therapeutic
^64^Cu	12.1 h	β+ = 0.653; β− = 0.578	Diagnostic PET; Therapeutic
^225^Ac	9.9 d	α = 8.40	Therapeutic

**Table 2:** Effect of changing molar-excess on the average number of DOTA chelators per antibody.

Molar Excess of p-SCN-Bn-DOTA	Average Number of Chelators Attached per Antibody
20-fold	3.00
40-fold	3.83
50-fold	4.50
100-fold	6.17

**Table 3:** Radiolabelling yield and radiochemical purity of radioimmunoconjugates.

Radioimmunoconjugate	Radiolabelling Yield (%) (ITLC)	Radiochemical Purity (%) (HPLC)
^99m^Tc-DOTA-C595	93	-
^68^Ga-DOTA-C595	50	-
^177^Lu-DOTA-C595	93	96
^64^Cu-DOTA-C595	>99	>99

**Table 4:** Cell binding results.

Radioimmunoconjugate Concentration (nM)	^177^Lu-DOTA-C595			^64^Cu-DOTA-C595		
	PANC-1 CPM (Mean ± S.D.)	AsPC-1 CPM (Mean ± S.D.)	*p*-Value	PANC-1 CPM (Mean ± S.D.)	AsPC-1 CPM (Mean ± S.D.)	*p*-Value
0	7.033 ± 39.11	−12.33 ± 16.70	0.474343	1704 ± 15.81	1644 ± 47.37	0.103428
10	1916 ± 1390	47.33 ± 14.30	0.080383	5515 ± 1880	3571 ± 560.0	0.160980
50	3723 ± 206.5	656.7 ± 767.9	0.013368 *	10,433 ± 3655	5167 ± 895.5	0.072480
100	5493 ± 771.7	962.7 ± 905.4	0.002738 **	16,846 ± 4819	7363 ± 2735	0.041383
200	13,293 ± 1078	2068 ± 1306	0.000328 ***	21,949 ± 5776	15,969 ± 4246	0.221960
300	21,337 ± 237.6	2867 ± 933.4	0.001357 **	24,477 ± 1505	16,613 ± 2259	0.007389
400	-	-	-	33,484 ± 4618	23,377 ± 4506	0.053370
500	24,354 ± 3485	5723 ± 2242	0.004905 **	38,833 ± 454.4	27,083 ± 7548	0.054576

* *p* < 0.05, ** *p* < 0.01, *** *p* < 0.001; CPM = counts per minute, S.D. = standard deviation.

**Table 5:** Coordination features of the radionuclides. Adapted from Price and Orvig [31].

Radionuclide	Charge	Coordination Number/Geometry	Ionic Radii (Å)
^99m^Tc	+4	VI, distorted octahedral	0.65
^68^Ga	+3	VI, distorted octahedral	0.62
^177^Lu	+3	VIII, square antiprism	0.98
^64^Cu	+2	VI, distorted octahedral	0.73

## Data Availability

The datasets used and/or analysed during the current study are available from the corresponding author on reasonable request.

## Supplementary Materials

The following supporting information can be downloaded at: https://www.mdpi.com/article/10.3390/cells11192983/s1, Figure S1: Examples of A, standard DOTA curve and B, standard protein curve used to quantify the molar ratio of p-SCN-Bn-DOTA to C595 antibody in the DOTA-C595 immunoconjugate; Figure S2: Difference in MUC1-CE expression across different pancreatic cancer cell lines. Image reused with permission from Hull et al. 22; Figure S3: Mass spectra of light chain p-SCN-Bn-DOTA-C595 conjugate stored in 0.1 M ammonium acetate (pH 6.4) at 37 °C at A, day 0 and B, day 7; Figure S4: Mass spectra of heavy chain p-SCN-Bn-DOTA-C595 conjugate stored in 0.1 M ammonium acetate (pH 6.4) at 37 °C at A, day 0 and B, day 7; Figure S5: Mass spectra of light chain p-SCN-Bn-DOTA-C595 conjugate stored in 0.1 M ammonium acetate (pH 7.4) at 37 °C at A, day 0 and B, day 7; Figure S6: Mass spectra of heavy chain p-SCN-Bn-DOTA-C595 conjugate stored in 0.1 M ammonium acetate (pH 7.4) at 37 °C at A, day 0 and B, day 7; Figure S7: Mass spectra of light chain p-SCN-Bn-DOTA-C595 conjugate stored in PBS (pH 7.4) at 4 °C at A, day 0 and B, day 30; Figure S8: Mass spectra of heavy chain p-SCN-Bn-DOTA-C595 conjugate stored in PBS (pH 7.4) at 4 °C at A, day 0 and B, day 30.
