# Supplementary material for: Preliminary Development and Testing of C595 Radioimmunoconjugates for Targeting MUC1 Cancer Epitopes in Pancreatic Ductal Adenocarcinoma
Source: Cells. 2022 Sep 24;11(19):2983. doi: 10.3390/cells11192983 (PMC9563759; doi:10.3390/cells11192983)
Supplement: Supplementary file 1 [file cells-11-02983-s001.zip › cells-1898539-supplementary.pdf]

Supporting Information

Preliminary development and testing of C595 radioimmunoconjugates for targeting MUC1 cancer epitopes in pancreatic ductal adenocarcinoma

Ashleigh Hull [1,2*], Yanrui Li [1], Dylan Bartholomeusz [2,3], William Hsieh [1,2], William Tieu [4], Tara Pukala [5], Alexander H. Staudacher [3,6] and Eva Bezak [1,5]

[1] Cancer Research Institute and Allied Health and Human Performance Academic Unit, University of South Australia, Adelaide SA 5001, Australia;

[2] Department of PET, Nuclear Medicine & Bone Densitometry, Royal Adelaide Hospital, SA Medical Imaging, Adelaide SA 5000, Australia.

[3] Adelaide Medical School, The University of Adelaide, Adelaide SA 5000, Australia.

[4] Molecular Imaging and Therapy Research Unit, South Australian Health and Medical Research Institute, Adelaide SA 5000, Australia.

[5] School of Physical Sciences, The University of Adelaide, Adelaide SA 5000, Australia.

[6] Translational Oncology Laboratory, Centre for Cancer Biology, SA Pathology and University of South Australia, Adelaide, SA 5000, Australia.

***** Correspondence: Ashleigh.hull@unisa.edu.au

Supporting information

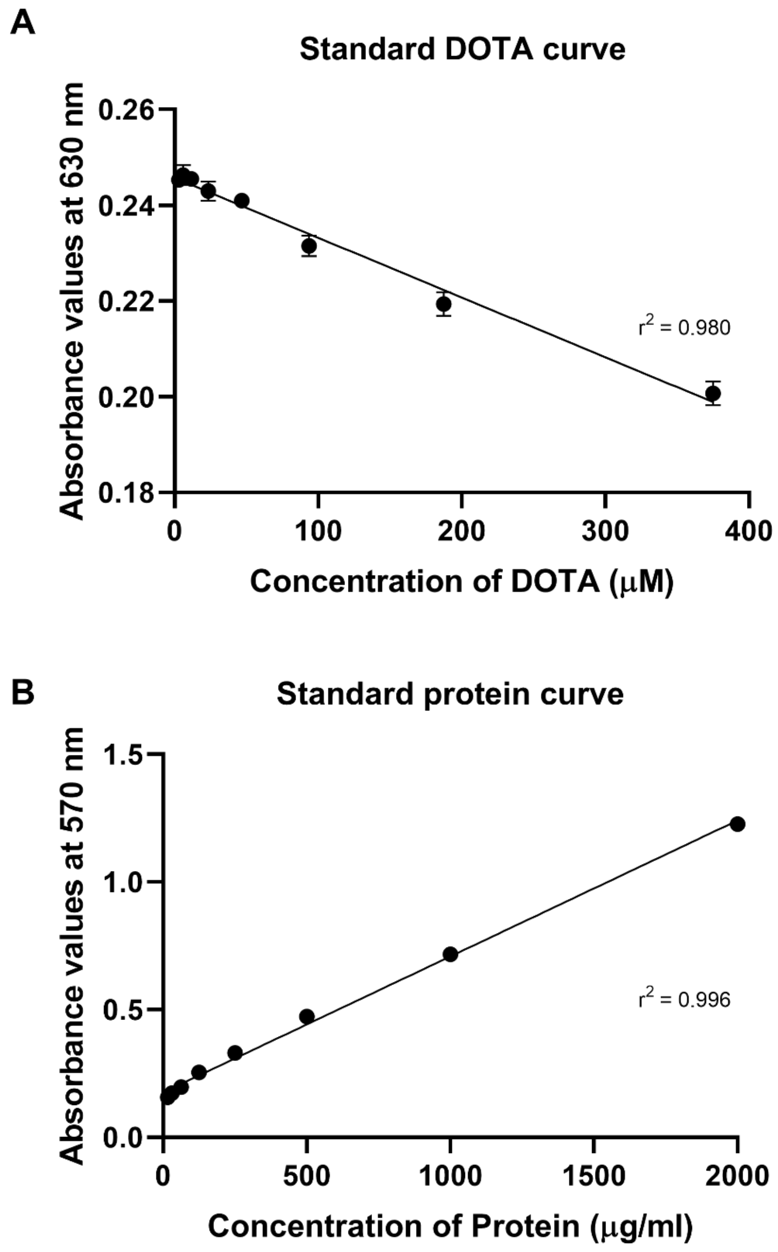

Figure S1: Examples of **A,** standard DOTA curve and **B,** standard protein curve used to quantify the molar ratio of p-SCN-Bn-DOTA to C595 antibody in the DOTA-C595 immunoconjugate.

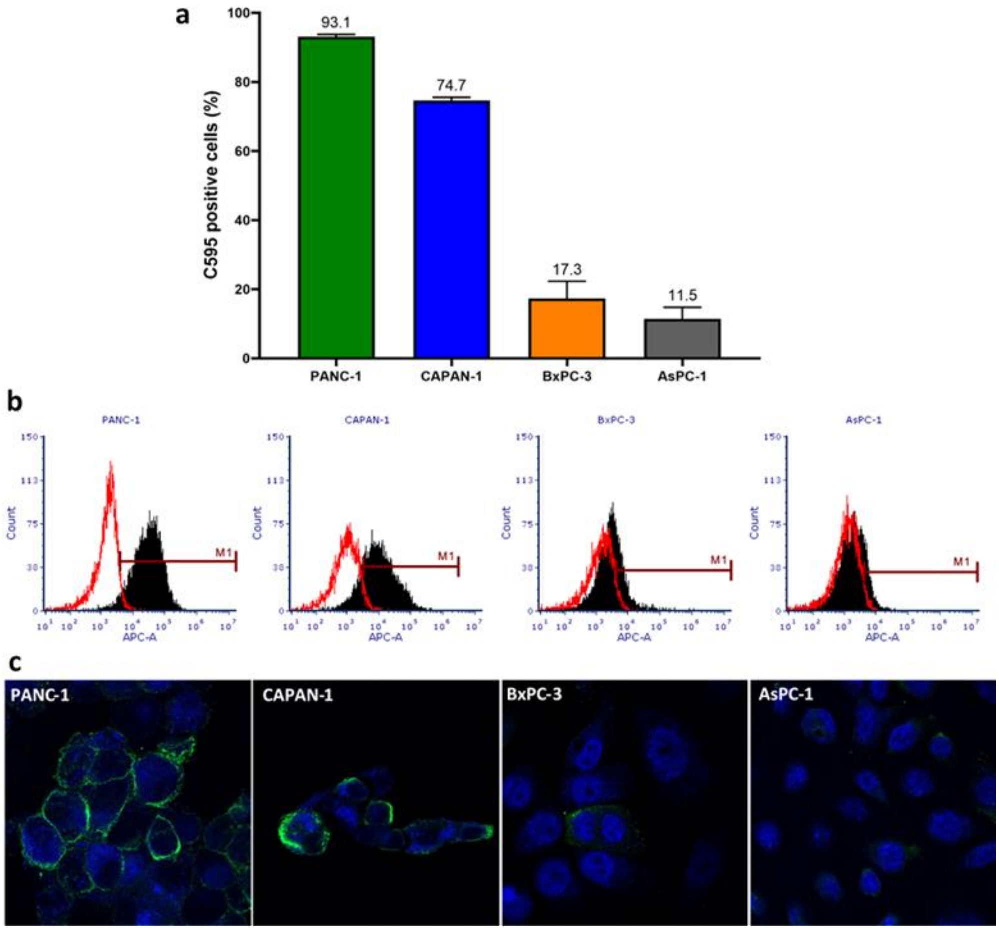

Figure S2: Difference in MUC1-CE expression across different pancreatic cancer cell lines. Image reused with permission from Hull et al. (*22*).

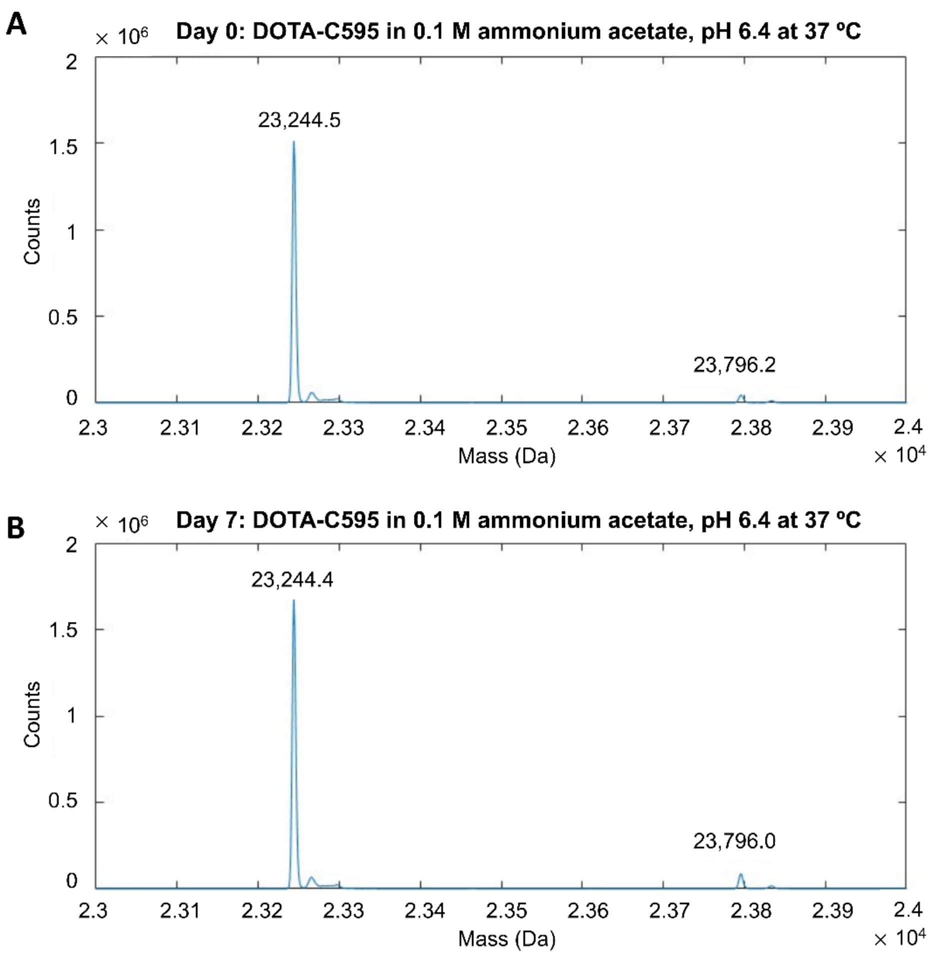

Figure S3: Mass spectra of light chain p-SCN-Bn-DOTA-C595 conjugate stored in 0.1M ammonium acetate (pH 6.4) at 37°C at **A,** day 0 and **B,** day 7.

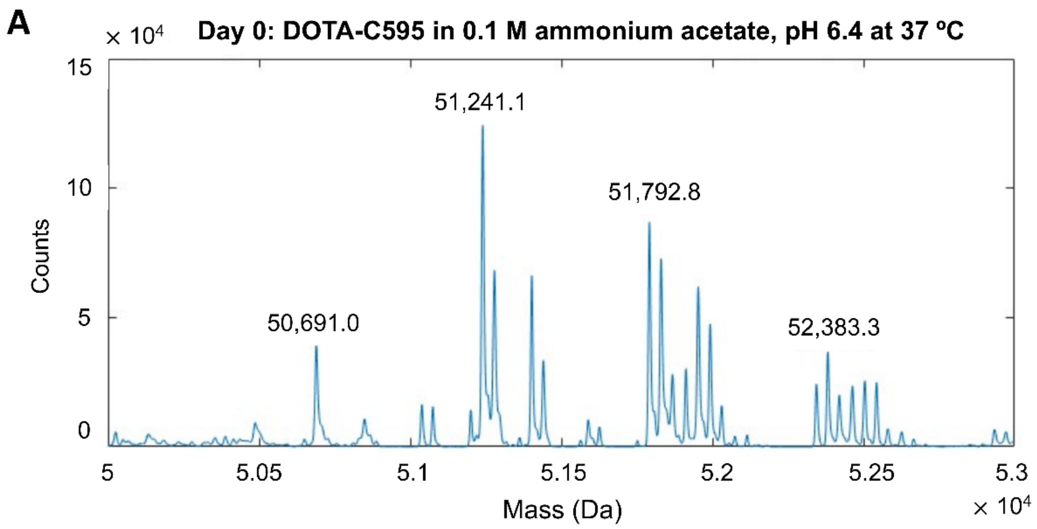

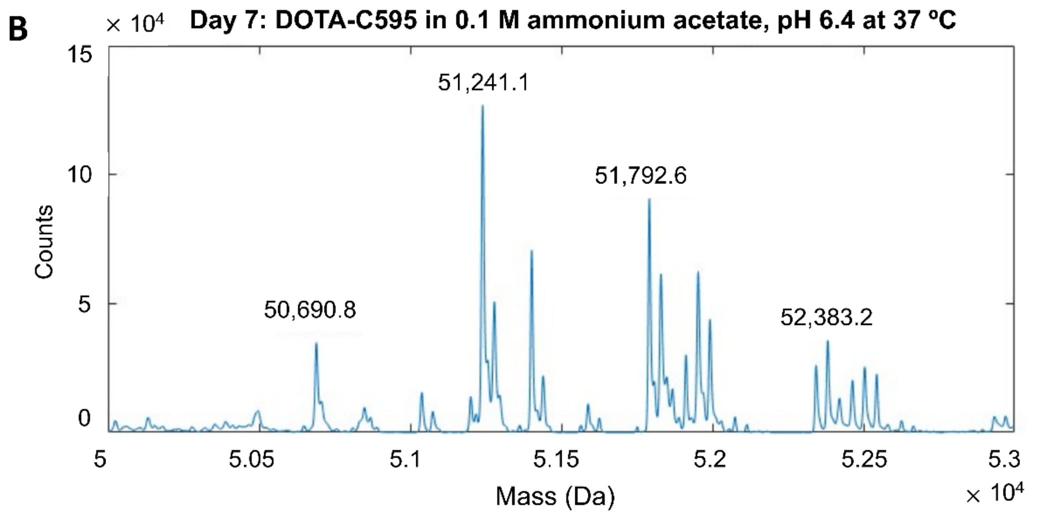

Figure S4: Mass spectra of heavy chain p-SCN-Bn-DOTA-C595 conjugate stored in 0.1M ammonium acetate (pH 6.4) at 37°C at **A,** day 0 and **B,** day 7.

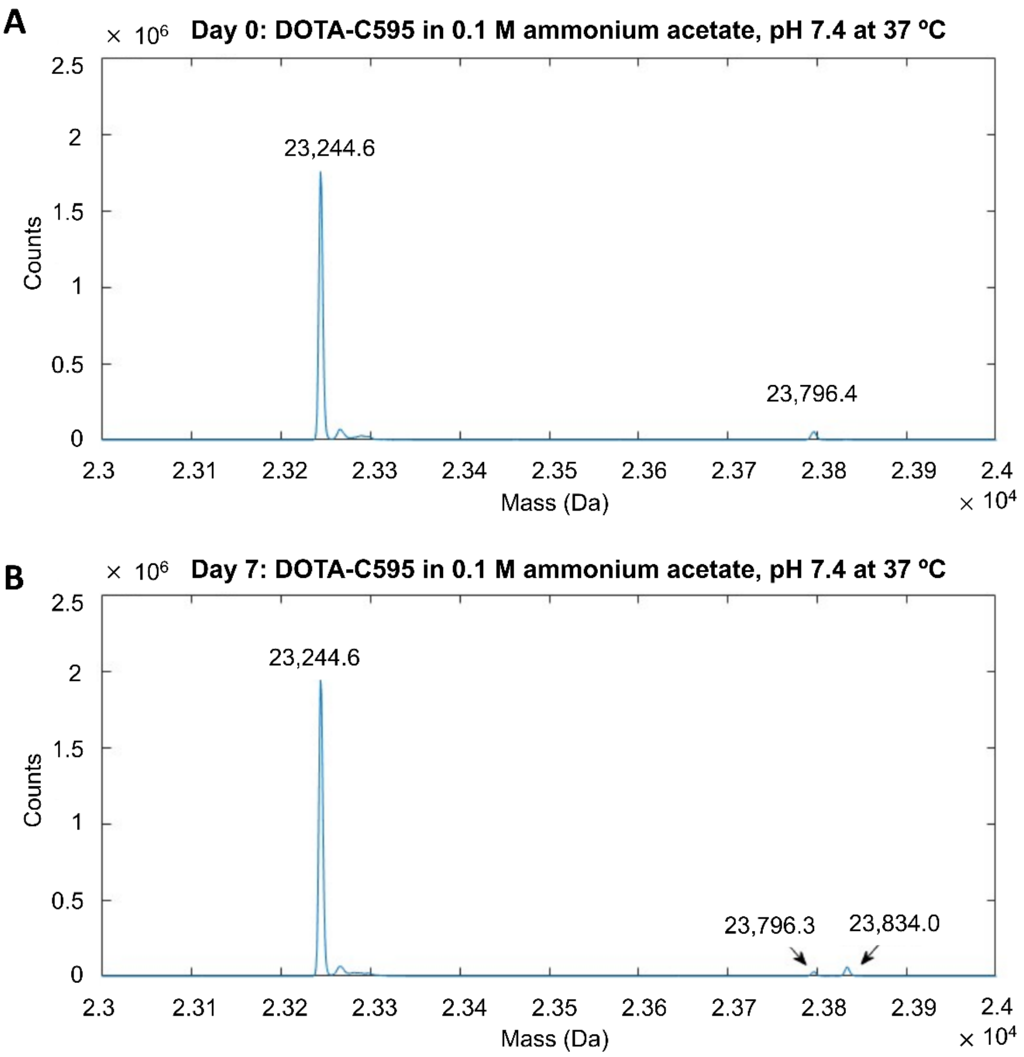

Figure S5: Mass spectra of light chain p-SCN-Bn-DOTA-C595 conjugate stored in 0.1M ammonium acetate (pH 7.4) at 37°C at **A,** day 0 and **B,** day 7.

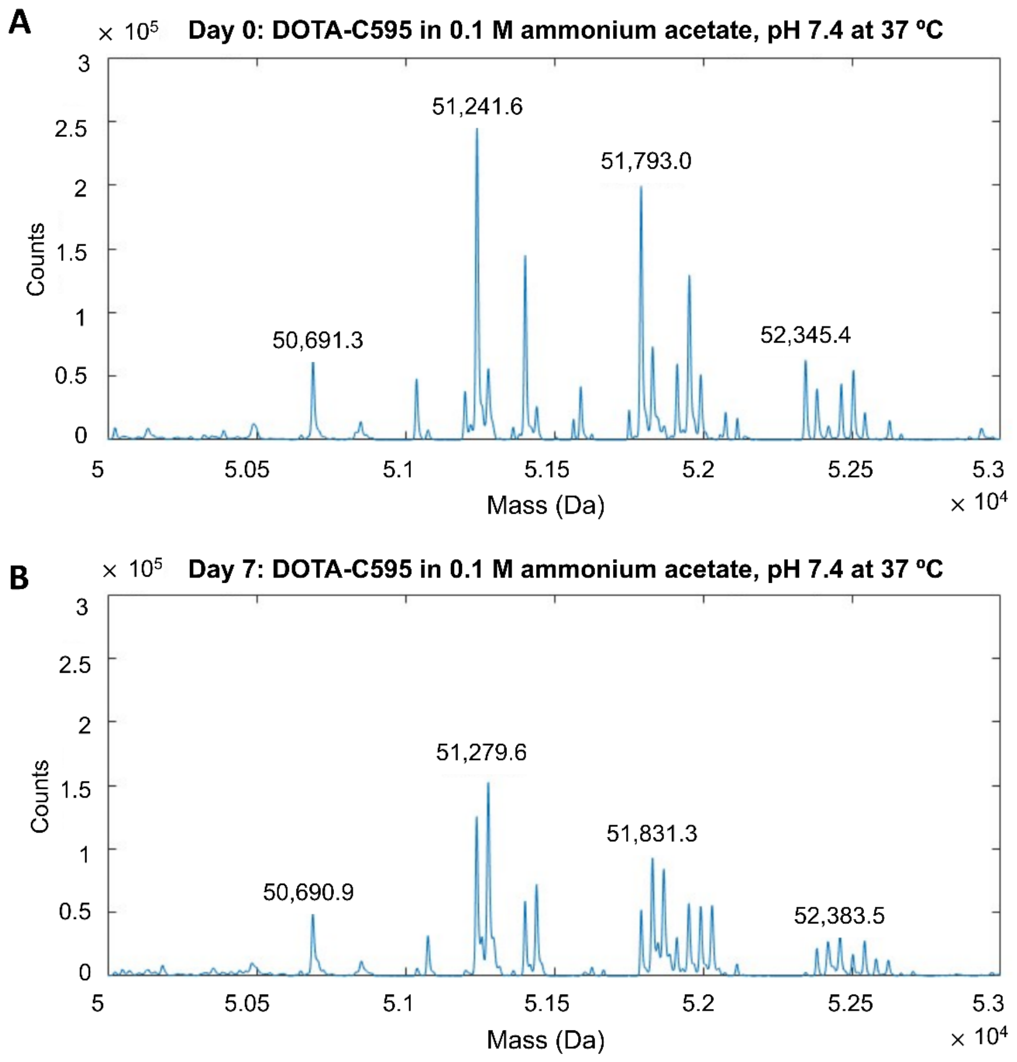

Figure S6: Mass spectra of heavy chain p-SCN-Bn-DOTA-C595 conjugate stored in 0.1M ammonium acetate (pH 7.4) at 37°C at **A,** day 0 and **B,** day 7.

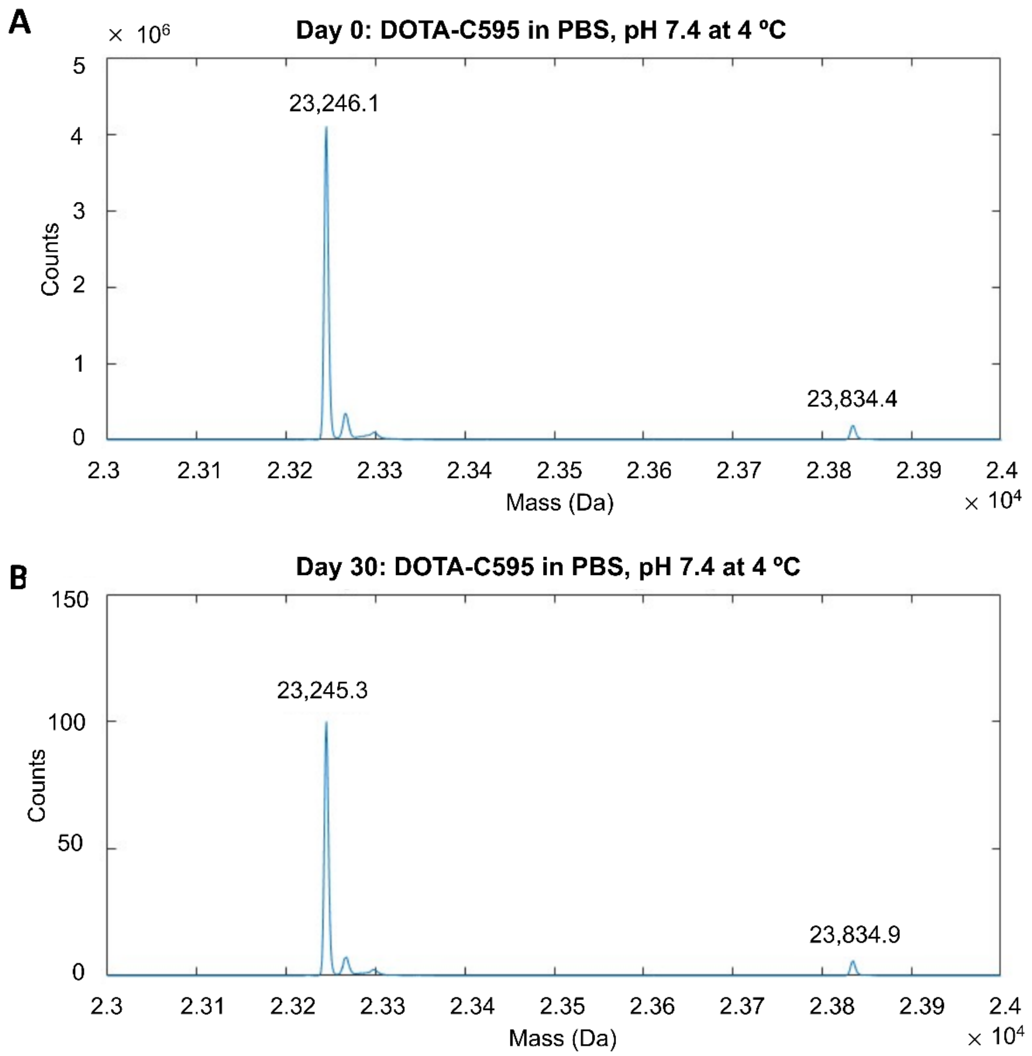

Figure S7: Mass spectra of light chain p-SCN-Bn-DOTA-C595 conjugate stored in PBS (pH 7.4) at 4°C at **A,** day 0 and **B,** day 30.

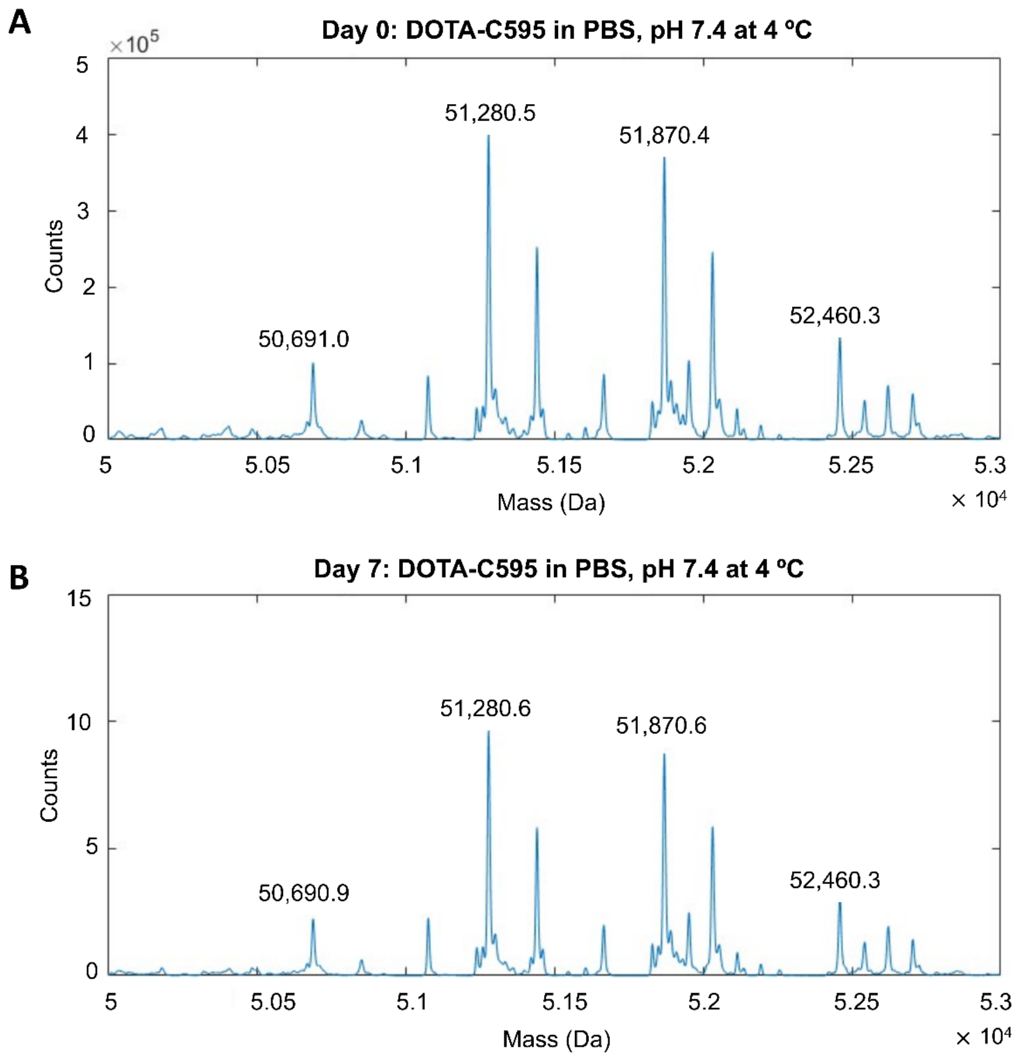

Figure S8: Mass spectra of heavy chain p-SCN-Bn-DOTA-C595 conjugate stored in PBS (pH 7.4) at 4°C at **A,** day 0 and **B,** day 30.
